# Connections between science and care within public and global health research: the need for attention and action

**DOI:** 10.7189/jogh.16.03017

**Published:** 2026-06-12

**Authors:** Martyn Pickersgill, Cathy Abbott, Harry Campbell, Anna Chiumento, Catherine J Crompton, Sue Fletcher-Watson, Charlotte Hanlon, Tracy Jackson, Felicity V Mehendale, Ting Shi, Rosie Stenhouse, Miranda R Waggoner, Sarah Wild

**Affiliations:** 1 School of Population Health Sciences, University of Edinburgh, Edinburgh, Scotland, UK; 2 School of Genetics and Cancer, University of Edinburgh, Edinburgh, Scotland, UK; 3 School of Social and Political Sciences, University of Edinburgh, Edinburgh, Scotland, UK; 4 School of Neurological and Cardiovascular Sciences, University of Edinburgh, Edinburgh, Scotland, UK; 5 School of Social Sciences, Rice University, Houston, Texas, USA

## Abstract

Connections between science and care are common, such as when people access particular therapies through participating in research. However, interactions go far beyond medical treatment *per se*, with other kinds of advice and support (including direct financial support) also warranting consideration. Such complexities of the science–care relationship are often not fully accounted for by formal ethical and governance processes and approvals. This can result in morally justifiable action, while also engendering uncertainty and unease among research teams. Acts that are ostensibly caring can on occasion and inadvertently also be harmful (*e.g.* the informal circulation of contested health-related claims or even ‘misinformation’ by research team members). Here, we argue that the connections between science and care require more dedicated attention from across the research ecosystem. This includes from funders, whose conventions can inappropriately restrict the care that researchers could and should provide. We also indicate the need for further studies by social scientists into how public and global health researchers navigate challenges related to care.

Science and care frequently connect. Take, for instance, participation within public and global health research. This has long been understood to be a key means through which populations might obtain treatments that may otherwise be inaccessible [1] and is increasingly recognised by and through legal and ethics scholars, committees, and norms. Such recognition has resulted in, for example, more frequent encouragements to provide ancillary care within health research conducted in resource-constrained settings [2].

More generally, interactions between science and care are part and parcel of health research, and can complicate conventional approaches to research ethics and good research practice. Scientists sometimes have to go ‘above and beyond’ to meet their own expectations of personal morality and integrity, and those of the communities with which they work. On occasion, they must renegotiate ethical frameworks in practice, in ways that ethical committees might not deem ideal, but which are nevertheless morally justifiable. These actions have their own social and ethical implications which demand attention and engagement, including from funders who would derive benefit from enhanced understandings of how their structures can both support and restrict science–care interactions.

## BEYOND FORMAL GOVERNANCE

If the ethical and legal governance of health research is taken for granted as a means of defining the specifically *medical* care that participating populations might receive, it can sometimes also generate friction with *other* means of caring for and with populations [3]. For example, well-intentioned assumptions by some ethics committees about the impropriety of engaging participants through the use of the online messaging platform WhatsApp can conflict with communities’ own conventions and communication preferences [4]. Default procedures in relation to post-study data anonymisation and deletion might also contribute to the systematic exclusion of some groups from having their data re-used in work that could maximise health benefits to them [5].

Likewise, failure to recognise the agency of individuals commonly judged to lack some kinds of capacity could result in so-called ‘vulnerable’ populations being overlooked as potential partners in collaborative, relational research [6]. This includes capacity for informed consent *per se* with respect to marginalised and stigmatised populations, where their exclusion from health research could itself extend injustice [7]. These issues are further complicated in cases where people experience coercion more generally, such as being involuntarily detained in their own homes or within mental health inpatient units, traditional or religious healing sites, and prisons.

There are also other norms and customs that impact health research beyond those formally imposed by ethical and regulatory approval bodies, and which shape how science and care connect [8]. In particular, funders themselves can inform what is deemed an ‘appropriate’ action in a given study. This is in part through what care they are prepared to resource and by what mechanisms (*e.g.* additional staff time), and what they are not. One implication is that grant recipients cannot always provide support to the communities with whom they work, or indeed to research team members who fall sick, to the degree that they regard as equitable and desirable. This in itself has implications for the production of knowledge: participants (and perhaps research team members, too) could come to disengage with specific projects or even an entire programme of connected research.

Of course, researchers might find workarounds to reduce the moral distress that is sometimes associated with not being funded to provide participants with diverse forms of care. From our own experiences and those of our collaborators, we know that these can be wide-ranging. They involve, but are not limited to, project investigators and staff working outside of their primary roles to help participants to navigate complex healthcare systems, to translate or provide additional health-related information, or to give emotional and (sometimes) financial support during difficult times.

For some research team members, such actions may fulfil values of compassion and/or charitable giving that align with religious or other ethical convictions. Nonetheless, acts of care might, in some cases, add to the unpaid labour of project staff – people who might already be working beyond capacity and/or be inadequately remunerated due to funder or institutional conventions. Accordingly, informal acts of compassion can on occasion further contribute to the stress of undertaking technically-demanding research within challenging timeframes [9].

Importantly, such acts potentially also present risks to participants themselves. We suggest that informal practices of care can also introduce the possibility of inaccurate or even unethical health-related or other advice from researchers. This is particularly pertinent when responding to the needs of already highly marginalised groups experiencing stigmatised conditions. There are also risks that apparently innocuous acts of care can be problematic; *e.g.*, the provision of certain types of snacks to people living with highly calibrated food plans [10].

## RECOMMENDATIONS

None of the issues highlighted above are straightforward to resolve. They involve, for instance, balancing contributions to research with fair benefit, as well as promoting fair employment practices with forms of work that are responsive to personal and community concerns and expressed needs. What we can do, though, is to encourage greater focus on them from across the research ecosystem, including from research funders themselves. We maintain that funders would benefit from close and open engagement with grant recipients to better appreciate their experiences of how science and care connect and interact – and with what ramifications. One example of this would be workshops and meetings that are not aimed at eliciting progress updates, but at trying to meaningfully understand what challenges researchers face when seeking to undertake morally as well as scientifically valorous research. Through doing so, funders might better be able to support those they sponsor, and the communities which are regarded as deriving benefit from population studies.

Part of this process of making visible the hidden challenges of acting ethically and doing good within population and global health must entail close social scientific examination of research-in-practice. This includes, for instance, open-minded sociological investigations of health research itself, as well as the open-mindedness of funders willing to support such work. Doing so will assist in illuminating the difficulties scientists face and help generate actionable insight that is sensitive to context while also sufficiently generalisable to have broad import. We encourage research communities, institutions, and funders to both support and to take seriously the understandings that might be derived from such scholarship, with a view to further enhancing the impact of public and global health research.
